# FMO5 Plays a Sex-specific Role in Goblet Cell Maturation and Mucus Barrier Formation

**DOI:** 10.1016/j.jcmgh.2025.101591

**Published:** 2025-07-19

**Authors:** Megan L. Schaller, Madeline M. Sykes, Joy Mecano, Sumeet Solanki, Wesley Huang, Ryan J. Rebernick, Safa Beydoun, Emily Wang, Amara Bugarin-Lapuz, Yatrik M. Shah, Scott F. Leiser

**Affiliations:** 1 Department of Molecular and Integrative Physiology, University of Michigan, Ann Arbor, Michigan; 2 Department of Molecular and Cellular Pathology, University of Michigan, Ann Arbor, Michigan; 3 Department of Cellular and Molecular Biology, University of Michigan, Ann Arbor, Michigan; 4 Medical Scientist Training Program, University of Michigan, Ann Arbor, Michigan; 5 Department of Computational Medicine and Bioinformatics, University of Michigan, Ann Arbor, Michigan; 6 Department of Internal Medicine, University of Michigan, Ann Arbor, Michigan

**Keywords:** ER Stress, Goblet Cells, Mucus Barrier, Sex-dependent

## Abstract

**Background & Aims:**

The intestine plays a key role in metabolism and nutrient and water absorption and provides both physical and immunological defense against dietary and luminal antigens. The protective mucosal lining in the intestine is a critical component of the intestinal barrier that, when compromised, can lead to increased permeability, a defining characteristic of inflammatory bowel disease, among other intestinal diseases. Here, we define a new role for the flavin-containing monooxygenase (FMO) family of enzymes in maintaining a healthy intestinal epithelium.

**Methods:**

Using *Caenorhabditis elegans*, we measure intestinal barrier function, actin expression, and intestinal damage response. In mice, we utilize an intestine-specific, tamoxifen-inducible knockout model of the mammalian homolog of *Cefmo-2*, *Fmo5*, and assess histology, mucus barrier thickness, and goblet cell physiology. We also treat mice with the endoplasmic reticulum (ER) chaperone tauroursodeoxycholic acid.

**Results:**

In nematodes, we find *Cefmo-2* is necessary and sufficient for intestinal barrier function and intestinal actin expression and is induced by intestinal damage. In mice, we find striking changes to the intestine within 2 weeks following FMO5 disruption. Alterations include sex-dependent changes in colon epithelial histology, goblet cell localization, and mucus barrier formation. These changes are significantly more severe in female mice, mirroring differences observed in patients with inflammatory bowel disease. Furthermore, we find increased protein folding stress in FMO5 knockout animals and successfully rescue the severe female phenotype with the addition of a chemical ER chaperone.

**Conclusions:**

Together, our results identify a highly conserved and novel role for FMO5 in the mammalian intestine and support a key role for FMO5 in maintenance of ER/protein homeostasis and proper mucus barrier formation.

## Graphical abstract

**Figure:**
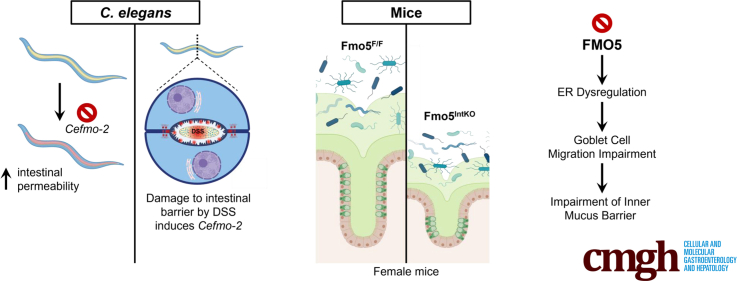

**Summary**

We find that flavin-containing monooxygenase 5 (FMO5) is necessary for crypt architecture and goblet cell function in the colonic epithelium of female mice. Female mice without FMO5 have impaired mucus production and barrier thickness, and we rescue these phenotypes with an endoplasmic reticulum chaperone.

This article has an accompanying Editorial.

The intestinal lining forms a tight barrier to dietary antigens and luminal microbes while also taking up nutrients, electrolytes, and water. The colonic epithelium is protected by a bi-layer of mucus directly adjacent to epithelial cells. The mucus barrier is composed of a dense inner layer with antimicrobial properties and a thick outer layer that is exposed to luminal contents.^1^ Mucus maintains the intestinal barrier and is made solely by cup-shaped goblet cells (GCs) abundant in the colonic epithelium. GCs constitutively synthesize mucins that, when secreted from storage vesicles, undergo biochemically induced structural changes to expand, hydrate, and form an interconnected structure that makes up the inner layer of the colonic mucosal lining.^2^, ^3^, ^4^ The absence of mucin-2 (MUC2), the primary mucin in colonic GCs, leads to altered crypt morphology and impairments in GC maturation and migration up the crypt.^5^ These changes are detrimental to intestinal homeostasis.

The continuous requirement for colonic mucus production to maintain the barrier underscores the importance of the endoplasmic reticulum (ER) and unfolded protein response (UPR) in GCs. The ER is required to efficiently fold the large MUC2 protein but also with promptly detecting and managing misfolded proteins. Impairments in GC ER function compromises the mucus barrier. Dysfunction in mucin processing and exocytosis from GCs is observed in patients with inflammatory bowel disease (IBD) (ulcerative colitis [UC]),^6^ negatively impacting mucus barrier function. Additionally, in mice with colitis and humans with UC, the inner mucus layer is infiltrated with microbes, suggesting a failure in the antimicrobial function of the mucus barrier.^7^ This increased permeability can result in inflammation, alterations in intestinal absorption, and damage to the intestinal epithelium.^7^^,^^8^

Flavin-containing monooxygenases (FMOs) are ER-resident enzymes that play an important role in xenobiotic metabolism.^9^^,^^10^ In previous studies, we discovered that *fmo-2* in *C. elegans* (*Cefmo-2*) is both necessary and sufficient to extend health and longevity following hypoxic and metabolic stress.^9^ *Cefmo-2* is primarily expressed in the intestine of *C. elegans*, and the FMO family is highly conserved across taxa.^11^ *Cefmo-2* is necessary to preserve health during environmental stress and aging in worms. The function, structure, and homeostatic pathways of the nematode intestine are largely conserved, making it a useful model organism to investigate intestinal integrity and stress.

Although the xenobiotic roles of FMOs have been well characterized, the endogenous roles of mammalian FMOs are still unclear.^12^^,^^13^ The mammalian homolog of *Cefmo-2*, *Fmo5*, is primarily expressed in epithelial cells of the small intestine and colon, as well as in hepatocytes.^14^^,^^15^ Flavin-containing monooxygenase 5 (FMO5) was recently reported as a regulator of metabolism and nutrient uptake using a constitutive knockout (KO) mouse model.^16^ However, little is known about the functional or cell autonomous role of FMO5 in the intestine. We developed an intestine-specific, tamoxifen-inducible FMO5 KO mouse to interrogate FMO function in the mammalian intestine. Here, through the establishment of this mouse model, we discovered that FMO5 plays a critical role in maintaining intestinal homeostasis. We show that disrupting FMO5 in the intestine of adult mice leads to aberrant GC localization and migration following 14 days of KO. We also find that intestinal FMO5 is necessary to maintain mucosal barrier thickness in mice, in a sex-dependent manner. We rescue these impairments with an ER stress chaperone, supporting a role for FMO5 in ER homeostasis. We propose a mechanistic role for FMO5 in potentiating mucin processing within the ER that is imperative for mucosal barrier homeostasis in mice.

## Results

### *fmo-2* Mediates Intestinal Integrity in *C. elegans*

**Figure 1:**
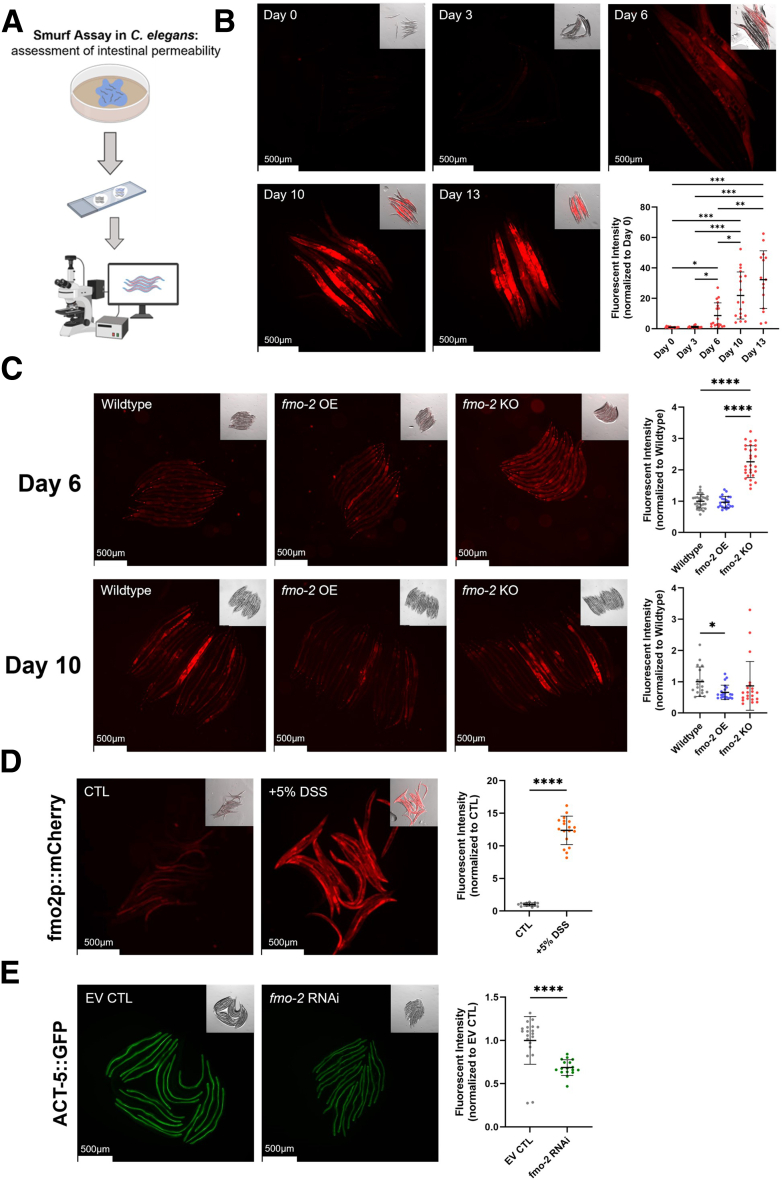
***fmo-2* mediates intestinal integrity and structure in *C*.***elegans*. (*A*) Diagram of the Smurf assay used to measure intestinal permeability in *C*. *elegans*. (*B*) Fluorescent images of wild-type (N2) worms fed Smurf-dyed (FD&C) food (*E. coli* OP50) at days 0, 3, 6, 10, and 13 of adulthood, and quantification of individual worm fluorescent intensity for each day (n = 15–18 worms/condition). (*C*) Smurf assay of wild-type, *fmo-2* OE, and *fmo-2* KO worms at days 6 (n = 10–16 worms/condition) and 10 of adulthood (n = 18–22 worms/condition), and quantification of individual worm fluorescent intensity for each day. (*D*) Fluorescent images of *fmo-2p::mCherry* worms on day 2 of adulthood following 20 hours of exposure to 5% DSS or M9 control, and quantification of individual worm fluorescent intensity (n = 14–18 worms/condition). (*E*) Fluorescent images of ACT-5::GFP worms on Empty Vector (EV) CTL or *fmo-2* RNAi on day 1 of adulthood, and quantification of individual worm fluorescent intensity (n = 17–20 worms/condition). Lines superimposed onto quantification graphs display mean ± SD normalized to Day 0, Wild-type, CTL, or EV CTL, respectively. Scale bars = 500 μm. Statistical significance for *B* and *C* was calculated using a 1-way ANOVA with Tukey correction for multiple comparisons. Significance for *D* and *E* was calculated by unpaired *t*-test, 2-tailed, with Welch’s correction. ^∗^*P* < .05, ^∗∗^*P* < .01, ^∗∗∗^*P* < .001, ^∗∗∗∗^*P* < .0001.

Based on previous work showing that *Cefmo-2* is necessary and sufficient to improve stress resistance and longevity and that it is expressed primarily in the intestine,^9^^,^^11^ we were interested whether *fmo-2* affects worm intestinal integrity. To assess intestinal permeability in *C. elegans*, we modified an assay commonly utilized in *Drosophila*, called the Smurf assay.^17^ This technique utilizes a nonabsorbable dye that does not cross the intestinal barrier in healthy organisms. Following intestinal injury in worms, the increased permeability leads to systemic detection of the fluorescent dye (Figure 1*A*). In wild-type worms, the intestinal permeability of *C. elegans* increases as the worm ages (Figure 1*B*). This finding is consistent with previous work in aged *C. elegans*, *D. melanogaster*, and mammals showing a gradual decline in intestinal integrity with age. To test whether *Cefmo-2* plays a role in maintaining intestinal integrity, we used the Smurf assay with *fmo-2* KO and *fmo-2* overexpressing (OE) worms. We found that worms without *fmo-2* display an increase in intestinal permeability by day 6 of adulthood (Figure 1*C*). By day 10 of adulthood, wild-type worms showed a similar increase in permeability as *fmo-2* KO worms, whereas *fmo-2* OE worms have less permeability than wild-type worms (Figure 1*C*). This result suggests that FMO-2 is critical in maintaining intestinal integrity during aging.

To test whether FMO-2 is also involved in responding to intestinal damage, we utilized dextran sodium sulfate (DSS) to induce intestinal injury.^18^ Employing a *fmo-2* transcriptional reporter worm strain, we find that *fmo-2* is significantly induced in worms following 20 hours of exposure to 5% DSS (Figure 1*D*). Combined with previously published results from our lab and others, where *fmo-2* is induced with exposure to other environmental stressors (dietary restriction, hypoxia, pathogens), this finding is consistent with a model of FMO-2 playing a role in broad environmental stress response.^9^^,^^19^ To assess the importance of *fmo-2* in maintaining the physical integrity of intestinal cells in *C. elegans*, we used an actin 5 (ACT-5) translational reporter worm strain and RNA interference (RNAi) to knockdown *fmo-2*. ACT-5 is the singular actin protein in the intestinal cells of *C. elegans*, where it makes up the structure of microvilli in the intestinal lumen.^20^ Knocking out ACT-5 in worms is embryonically lethal, and the amount of ACT-5 is directly related to the integrity of intestinal cells, where lower levels of ACT-5 lead to increased permeability.^20^ We find significantly decreased levels of ACT-5 protein in worms when *fmo-2* is knocked down with RNAi (Figure 1*E*), suggesting *fmo-2* is necessary for maintaining intestinal integrity in the basal state.

### FMO5 Plays a Sex-dependent Role in Maintaining Intestinal Crypt Architecture

**Figure 2:**
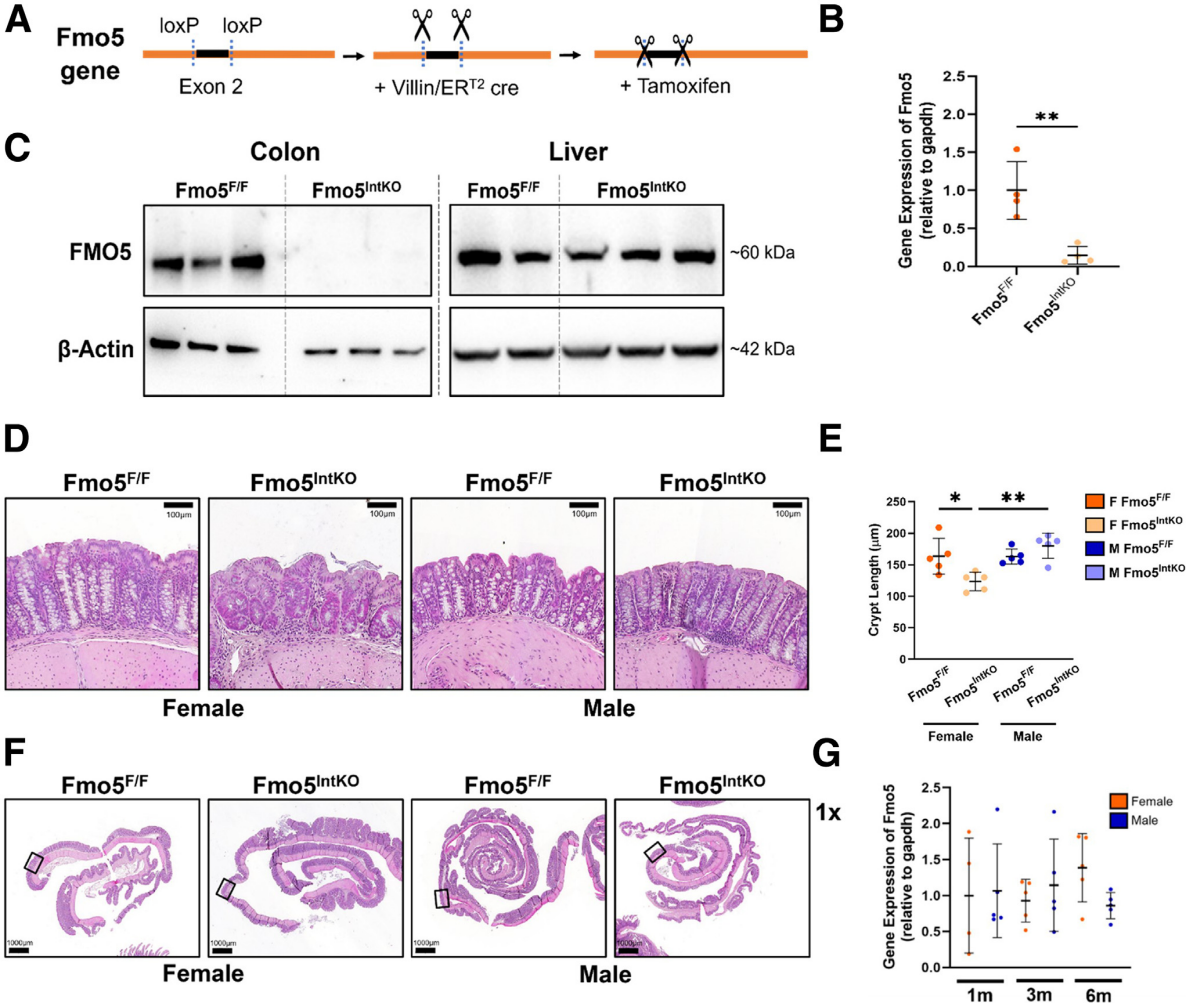
**FMO5 maintains crypt architecture in female mice.** (*A*) Diagram depicting the design of the VillinER^T2^ cre^+^; Fmo5^loxP/loxP^ mouse line. (*B*) Relative gene expression of *Fmo5* in colonic epithelium in Fmo5^F/F^ and Fmo5^IntKO^ mice 14 days after tamoxifen treatment (n = 4–5 mice/group). Lines superimposed onto plot display mean ± SD. Statistical significance between groups was calculated using an unpaired *t*-test, 2-tailed. (*C*) Western blot of FMO5 and β-Actin proteins in colon epithelium and liver of Fmo5^F/F^ and Fmo5^IntKO^ mice at 14 days of KO. (*D*) Representative H&E-stained images of the proximal colon in female and male Fmo5^F/F^ and Fmo5^IntKO^ mice after 14 days of KO. 20× magnification, Scale bar = 100 μm. (*E*) Crypt length quantification (μm) of female and male Fmo5^F/F^ and Fmo5^IntKO^ mice proximal colon epithelium represented in *D*. Each data point represents the average length of 40 to 50 crypts measured within an individual mouse (n = 4–5 mice/group). (*F*) Representative Swiss roll images of the proximal half of colons stained for H&E, corresponding to panel *D*. Boxes indicate the regions from which the representative images in panel *D* were taken. 1× magnification, Scale bar = 1000 μm. (*G*) Relative gene expression of *Fmo5* from colon epithelium of 1-, 3-, and 6-month old female and male wild-type mice (n = 4–5 mice/group). A nested 1-way ANOVA with multiple comparisons and Tukey post-hoc correction was performed for data in *E* and *G* to determine statistical significance. Significant differences between groups are represented by: ^∗^*P* < .05, ^∗∗^*P* < .01.

There are 5 FMOs in *C. elegans* and mammals. The phylogenic analysis of active site sequences of FMO proteins suggests that all *C. elegans* FMOs are most closely related to mammalian FMO5.^21^ FMO5 in mammals is primarily found in the liver and the epithelium of the small intestine and colon.^15^ To explore the conserved role of *Cefmo-2*/FMO5 we generated a FMO5 floxed mouse line (Fmo5^F/F^). Building on our finding that *fmo-2* is necessary for a healthy intestine in *C. elegans*, we crossed the FMO5 floxed mouse line with an intestinal epithelium-specific, tamoxifen-inducible VillinER^T2^ cre line (Fmo5^IntKO^) (Figure 2*A*). Gene expression in colonic epithelium and Western protein analysis 2 weeks after FMO5 disruption revealed significantly less FMO5 expression (Figure 2*B*) and protein levels (Figure 2*C*) in the colonic epithelium of Fmo5^IntKO^ mice when compared with Fmo5^F/F^ mice. FMO5 protein levels were maintained in the liver of Fmo5^IntKO^ mice, validating the tissue specificity of this knockout model (Figure 2*C*).

Histologic analysis of the colonic epithelium in these mice revealed significantly shorter crypts and a dysregulation in the uniformity of crypt structure at 14 days of KO in the proximal colon of female Fmo5^IntKO^ mice, as compared with their Fmo5^F/F^ littermates (Figure 2*D–F*). In male Fmo5^IntKO^ mice, we found elongated crypts compared with male Fmo5^F/F^ mice (Figure 2*D–F*). This phenotype occurred despite no differences in colonic *Fmo5* expression between female and male wild-type mice (Figure 2*G*). Sex differences have been noted in several gastrointestinal diseases in humans.^22^

### Crypt Uniformity Progressively Declines Over 14 Days After Intestinal FMO5 KO in Female Mice

**Figure 3:**
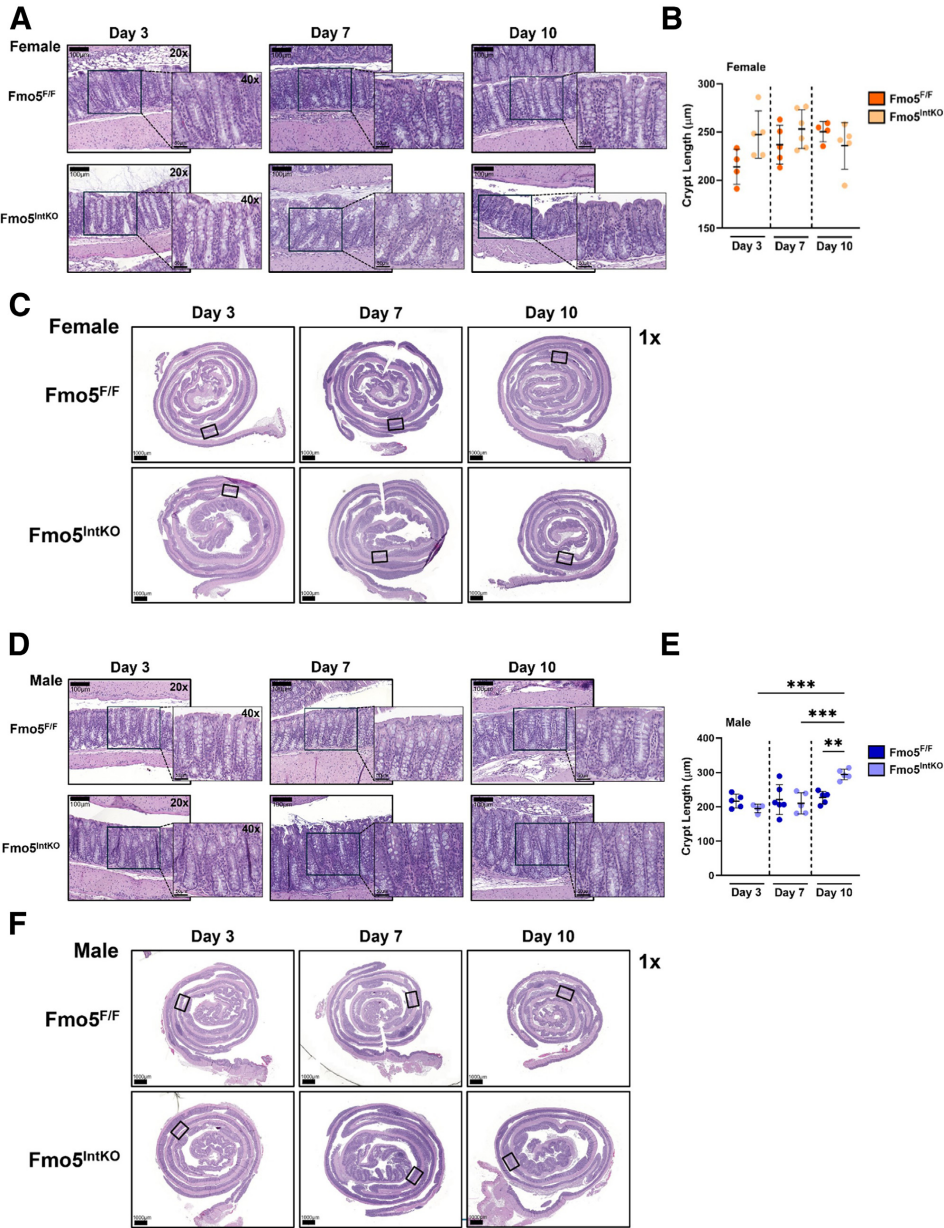
**Time course of FMO5 KO effects on crypt architecture.** (*A, D*) Representative H&E-stained images of the proximal colon from female (*A*) and male (*D*) Fmo5^F/F^ and Fmo5^IntKO^ mice at days 3, 7, and 10 of KO. Scale bar = 100 μm, 20× magnification. Inset images display each boxed area at 40× magnification. Scale bar = 50 μm. (*B, E*) Crypt length quantification (μm) of female (*B*) and male (*E*) mice represented in *A* and *D*. Each data point represents the average length of 40 to 50 crypts measured within an individual mouse (n = 4–6 mice/group), with error bars displaying mean +/- SD. (*C, F*) Representative Swiss roll images of the whole colon stained for H&E, corresponding to panels *A* and *D*. Boxes indicate the regions from which the representative images in panels *A* and *D* were taken. 1× magnification, Scale bar = 1000 μm. A nested 1-way ANOVA with multiple comparisons and Tukey post-hoc correction was performed for data in *B* and *E* to determine statistical significance. ^∗∗^*P* < .01, ^∗∗∗^*P* < .001.

To determine if FMO5 plays a role in maintaining crypt architecture and/or if FMO5 is necessary for epithelial cell turnover in the colon, we performed hematoxylin and eosin (H&E) staining in Fmo5^IntKO^ and Fmo5^F/F^ mice at 3, 7, and 10 days following tamoxifen treatment. After 3 days of KO, we did not see distinct differences in epithelial architecture between female Fmo5^IntKO^ and Fmo5^F/F^ mice (Figure 3*A–C*). However, 7 days following the disruption of FMO5 in the intestine, there was a loss of crypt uniformity in female Fmo5^IntKO^ mice (Figure 3*A*). Crypts were stacked on top of one another, causing the epithelium to appear increasingly disorganized. At day 10 of KO, the uniformity of crypt architecture remained lost in these mice (Figure 3*A*).

Similar to what we observed in females, there were no differences between male Fmo5^F/F^ and Fmo5^IntKO^ mice at day 3 and 7 of KO (Figure 3*D–F*). At 10 days post KO, male Fmo5^IntKO^ mice had significantly longer crypts in the proximal colon than both their wild-type littermates and Fmo5^IntKO^ mice at days 3 and 7 (Figure 3*D, E*). The absence of significant epithelial changes in both female and male Fmo5^IntKO^ mice after 3 days indicates that FMO5 is not essential for acute cell survival but rather affects the subsequent turnover and development of the epithelium.

### Female Fmo5^IntKO^ Mice Have Impaired GC Localization

**Figure 4:**
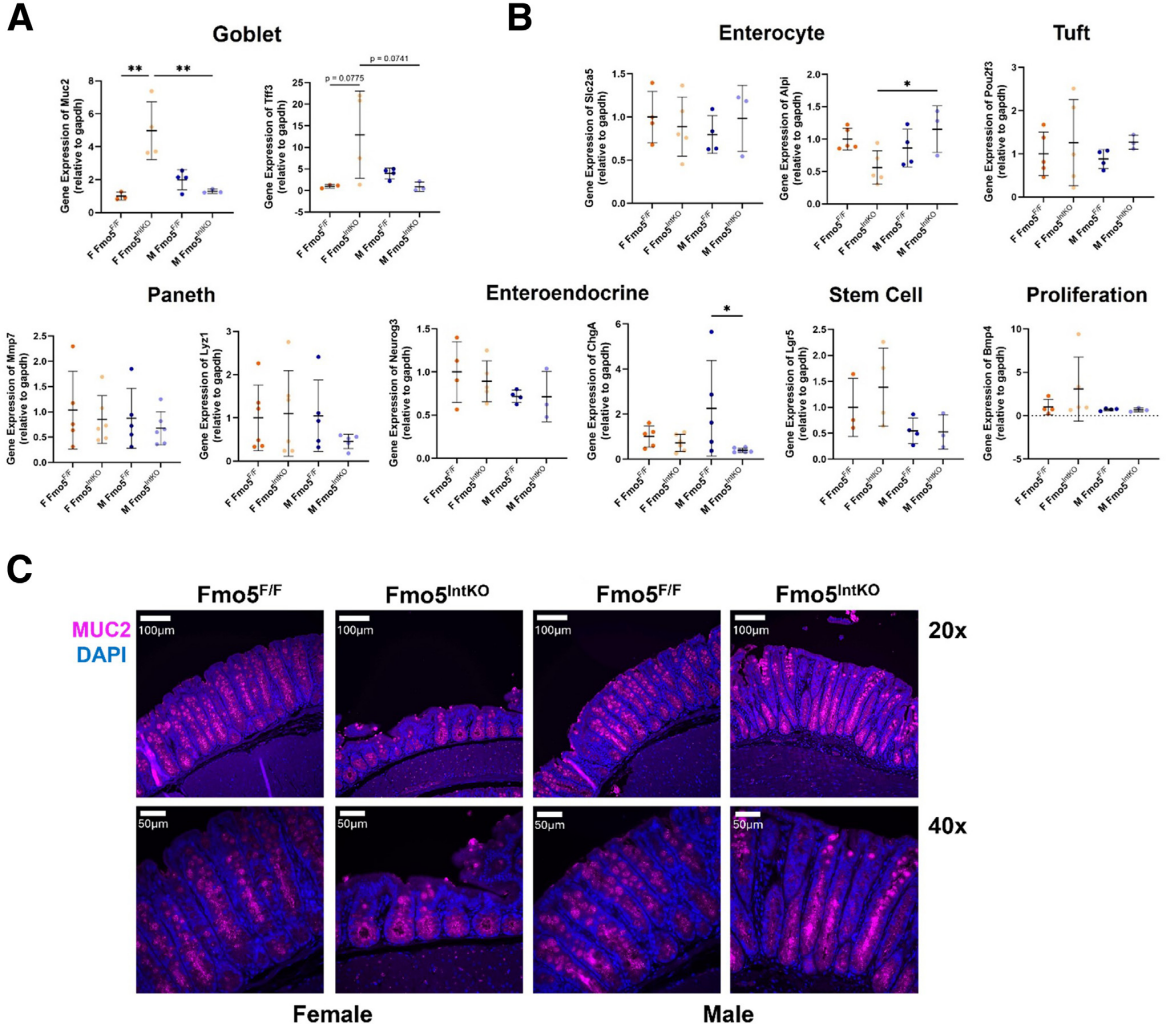
**Loss of FMO5 impairs goblet cell expression and localization.** (*A*) Relative gene expression of *Muc2* and *Tff3* in colonic epithelium of female (F) and male (M) Fmo5^F/F^ and Fmo5^IntKO^ mice 14 days after tamoxifen treatment (n = 3–4 mice/group). Lines superimposed onto plot display mean ± SD. (*B*) Relative gene expression of intestinal cell type markers *Slc2a5*, *Alpi*, *Mmp7*, *Lyz1*, *Pou2f3*, *Neurog3*, *ChgA*, *Lgr5*, and *Bmp4* from the colonic epithelium of mice described in *A*. (*C*) Representative images of IF staining of MUC2 (*magenta*) and DAPI (*blue*) in female and male Fmo5^F/F^ and Fmo5^IntKO^ proximal colons at day 14 of KO. Scale bar = 100 μm, 20× magnification. Scale bar = 50 μm, 40× magnification. A nested 1-way ANOVA with multiple comparisons and Tukey post-hoc correction was performed for data in *A* and *B*. Significant differences between groups are represented by: ^∗^*P* < .05, ^∗∗^*P* < .01.

The intestinal epithelium is made up of a heterogenous population of epithelial cells including enteroendocrine, absorptive, and secretory cell types, each performing distinct functions to maintain homeostasis.^23^^,^^24^ We found that the expression of the GC gene *Muc2* was significantly increased in female Fmo5^IntKO^ mice (Figure 4*A*). Also, trefoil factor 3 (*Tff3*) was increased but not statistically significant, and this was not observed in male Fmo5^IntKO^ mice (Figure 4*A*). We did not find significant differences in absorptive enterocyte (solute carrier family 2 member 5 [*Slc2a5*] and alkaline phosphatase intestinal [*Alpi*]), Paneth (matrix metallopeptidase 7 [*Mmp7*] and *lysozyme-like protein 1* [*Lyz1*]), tuft (POU class 2 homeobox 3 [*Pou2f3*]), enteroendocrine (neurogenin 3 [*Neurog3*] and chromogranin A [*ChgA*]), stem cell (leucine-rich repeat containing G protein-coupled receptor 5 [*Lgr5*]), or proliferation (bone morphogenetic protein 4 [*Bmp4*]) markers (Figure 4*B*). Immunofluorescence (IF) staining of MUC2 (magenta) revealed altered localization of GCs in female Fmo5^IntKO^ mice, as GCs are clustered at the bottom of the crypt compared to Fmo5^F/F^ mice, where GCs are evenly distributed throughout the crypt (Figure 4*C*).

### Female Fmo5^IntKO^ Mice Have Impaired GC Migration

**Figure 5:**
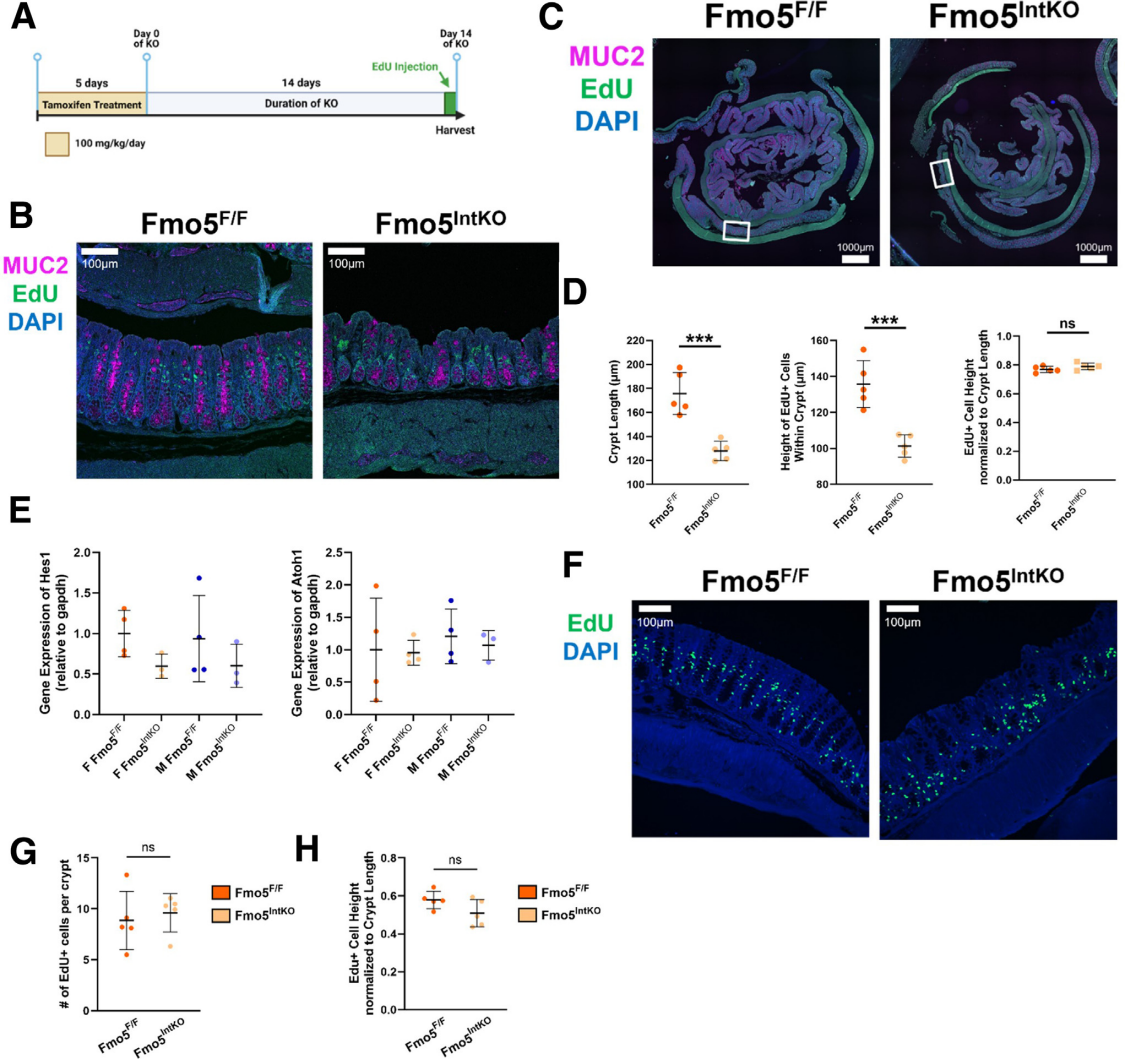
**Loss of FMO5 impairs GC migration.** (*A*) Experimental design of tamoxifen treatment and EdU labeling injection. (*B*) Representative images of MUC2 (*magenta*), EdU (*green*), and DAPI (*blue*) stained proximal colons from female Fmo5^F/F^ and Fmo5^IntKO^ mice collected 24 hours after EdU labeling. Scale bar = 100 μm, 20× magnification. (*C*) Representative Swiss roll images of the proximal colon stained for MUC2 (*magenta*), EdU (*green*), and DAPI (*blue*), corresponding to panel *B*. Boxes indicate the regions from which the representative images in panel *B* were taken. 4× magnification, Scale bar = 1000 μm. (*D*) Quantification of crypt length (μm) and the respective height of EdU+ cells within that crypt 24 hours after EdU labeling (day 14 of KO). Quantified EdU+ cell migration height (μm) normalized by each specific crypts’ length (μm). Each data point is the average of 10 to 25 crypts measured within each mouse (n = 5 mice/group), with error bars displaying mean ± SD of measurements from that group. (*E*) Relative gene expression of absorptive and secretory progenitor cell markers *Hes1* and *Atoh1* from colonic epithelium of ∼10-week-old female (F) and male (M) Fmo5^F/F^ and Fmo5^IntKO^ mice (n = 3–6 mice/group). Plots display mean ± SD for each group. (*F*) Representative images of EdU (*green*) and DAPI (*blue*) stained proximal colons collected from female Fmo5^F/F^ and Fmo5^IntKO^ mice 2 hours after EdU labeling. (*G, H*) Quantification of the average number of EdU+ cells per crypt (*G*) and the starting position of EdU+ cell height normalized to individual crypt length (*H*) 2 hours post EdU injection, as depicted in *F*. Unpaired *t*-tests were used to calculate statistical significance between groups in *D*, *G*, and *H*. A nested 1-way ANOVA with multiple comparisons and Tukey post-hoc correction was performed for data in *E*. ns = no significant difference, ^∗∗∗^*P* < .001.

To determine if female Fmo5^IntKO^ mice have an impairment in overall epithelial cell migration, we performed a pulse-chase experiment in female Fmo5^F/F^ and Fmo5^IntKO^ mice with 5-ethynyl-2’-deoxyuridine (EdU), for 24 hours before collecting the colon for histologic analysis (Figure 5*A*). We then performed IF staining of MUC2 (magenta) and EdU (green) (Figure 5*B, C*) to visualize the location of cells that were actively replicating when EdU was injected 24 hours prior. We measured the distance up the crypt that the EdU+ cells had migrated within 24 hours and normalized that distance to individual crypt length, finding no difference in the migration distance of EdU+ cells between female Fmo5^F/F^ and Fmo5^IntKO^ mice at 24 hours (Figure 5*D*). This supports the conclusion that overall epithelial cell migration is not altered in female Fmo5^IntKO^ mice, but that there is a migration impairment specific to GCs. GCs mature as they migrate up the crypt towards the lumen of the intestine, which is required to properly secrete mucin in response to systemic cues.^25^^,^^26^ In support of this, we found no difference in absorptive or secretory progenitor cell expression (hes family bHLH transcription factor 1 [*Hes1*], atonal bHLH transcription factor 1 [*Atoh1*]) in our mice (Figure 5*E*), suggesting the lack of GCs in the upper crypt is not due to a decrease in secretory cell lineage differentiation, but rather a possible impairment in GC maturation. To assess proliferation, we performed an additional pulse-chase experiment in female Fmo5^F/F^ and Fmo5^IntKO^ mice with EdU and harvested the colon 2 hours post EdU injection. We found no difference in the average number of EdU+ cells per crypt between female Fmo5^F/F^ and Fmo5^IntKO^ mice (Figure 5*F, G*), indicating that proliferation differences are not likely a major contributing factor to the crypt architecture defect in female Fmo5^IntKO^ mice. To determine whether FMO5 loss affects the initial positioning of cells within the crypt before migration, we measured the height of EdU+ cells in the crypts of female Fmo5^F/F^ and Fmo5^IntKO^ mice at 2 hours post EdU injection, normalizing to crypt length. We found no significant differences in the initial positioning of EdU+ cells between genotypes (Figure 5*F, H*). These results, in conjunction with the spatial impairments of GCs within crypts observed in female Fmo5^IntKO^ mice, support a role for FMO5 in GC development and maturation.

### FMO5 Is Necessary for Mucus Production and Barrier Thickness in Female Mice

**Figure 6:**
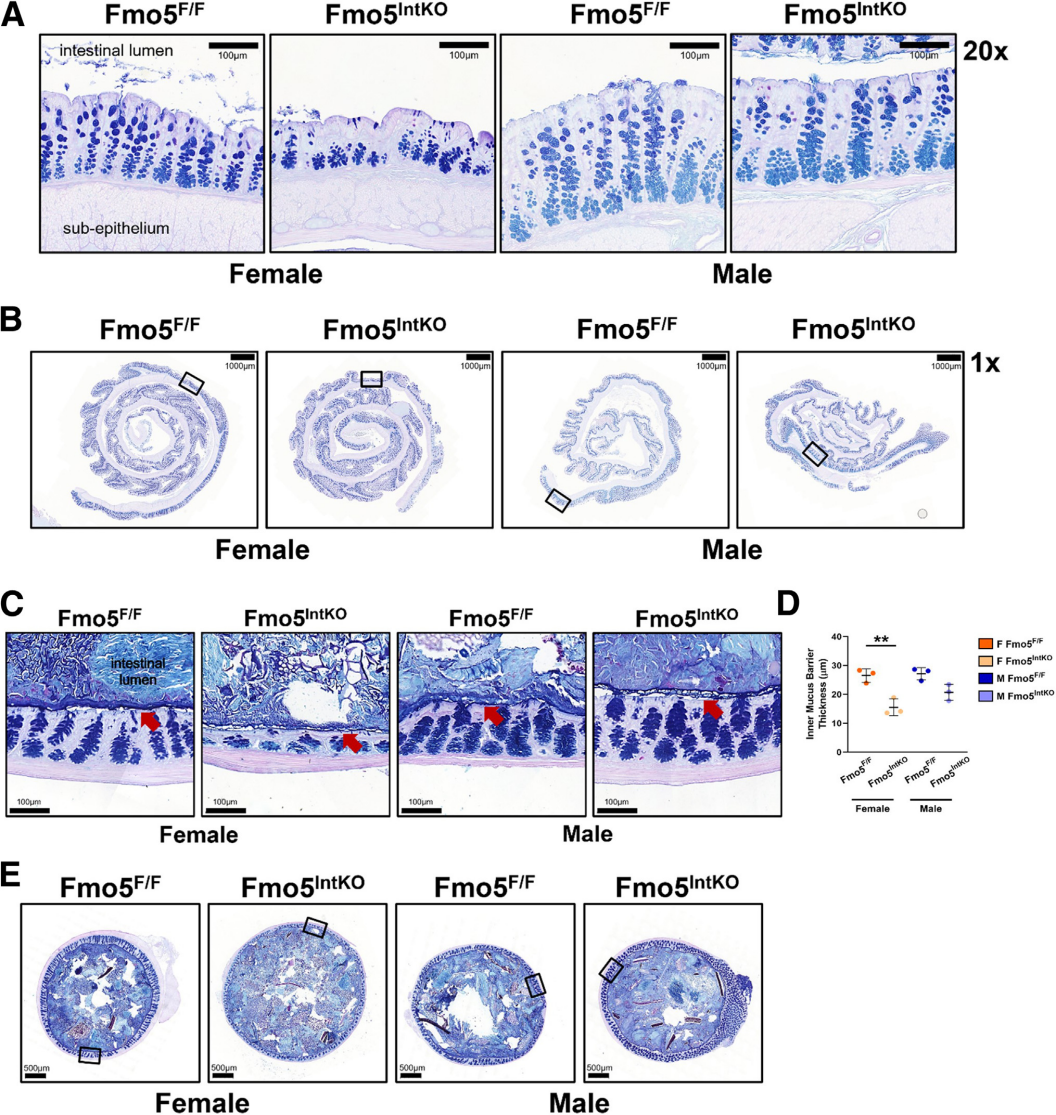
**Loss of FMO5 decreases mucus barrier thickness in female mice.** (*A*) Representative AB/PAS-stained images from the proximal half of the colon of female and male Fmo5^F/F^ and Fmo5^IntKO^ mice at day 14 of KO. (*B*) Representative Swiss roll images of the proximal colon stained for AB/PAS, corresponding to panel *A*. Boxes indicate the regions from which the representative images in panel *A* were taken. 1× magnification, Scale bar = 1000 μm. (*C*) Representative images of colon cross-sections stained with AB/PAS and processed using Carnoy’s fixative to preserve luminal mucus barrier in female and male Fmo5^F/F^ and Fmo5^IntKO^ mice 14 days following tamoxifen treatment, 20× magnification. Scale bar = 100 μm. *Red arrows* point to the inner mucus barrier. (*D*) Quantified inner mucus barrier thickness (μm) of images represented in *C*. Each data point is an average of ∼30 measurements of inner mucus barrier thickness within each individual mouse. Error bars display mean ± SD for each mouse. (*E*) Representative cross-sectional images of the colon stained for AB/PAS, corresponding to panel *C*. Boxes indicate the regions from which the representative images in panel *C* were taken. 2× magnification, Scale bar = 500 μm. Statistical significance in *D* was calculated using nested 1-way ANOVA with multiple comparisons and Tukey post-hoc correction. ^∗∗^*P* < .01.

MUC2 protein undergoes complex folding and N-glycosylation within the ER^27^ before moving to the Golgi apparatus to be O-glycosylated.^28^ Following post-translational modification, mucin is stored in secretory vesicles in the cup-shaped region of the cell to await release into the crypt or luminal space. Given that GC localization and maturity is impaired in female Fmo5^IntKO^ mice, we next wanted to test the functional capacity of GCs to produce mucus. Alcian blue/periodic acid–Schiff’s reagent (AB/PAS) staining of mucins within the crypts of Fmo5^F/F^ and Fmo5^IntKO^ mice revealed an overall decrease in stained mucins within the crypts of female Fmo5^IntKO^ mice (Figure 6*A, B*). This suggests that the spatial and migration impairments of GCs in these mice are sufficient to negatively impact their capacity to produce mucins within the crypt. We observed a slight reduction in AB/PAS-stained mucins in some crypts from male Fmo5^IntKO^ mice (Figure 6*A, B*) at 14 days of KO, although not to the severity observed in females. To determine the impact of acute FMO5 loss on mucus barrier formation, we used Carnoy’s fixative solution to preserve the mucus barrier during the processing of colon cross-sections (Figure 6*C, E*). With AB/PAS staining, we find a significantly thinner inner mucus layer in female Fmo5^IntKO^ mice at day 14 of KO when compared with female Fmo5^F/F^ mice (Figure 6*C–E*). Overall, these results demonstrate a loss of mucin-filled GCs within the crypts of female Fmo5^IntKO^ mice and establish the necessity of FMO5 to maintain mucus barrier thickness in mice.

### FMO5 Is Necessary to Maintain Uniformity in Mucin-filled Crypt GCs During Epithelial Turnover

**Figure 7:**
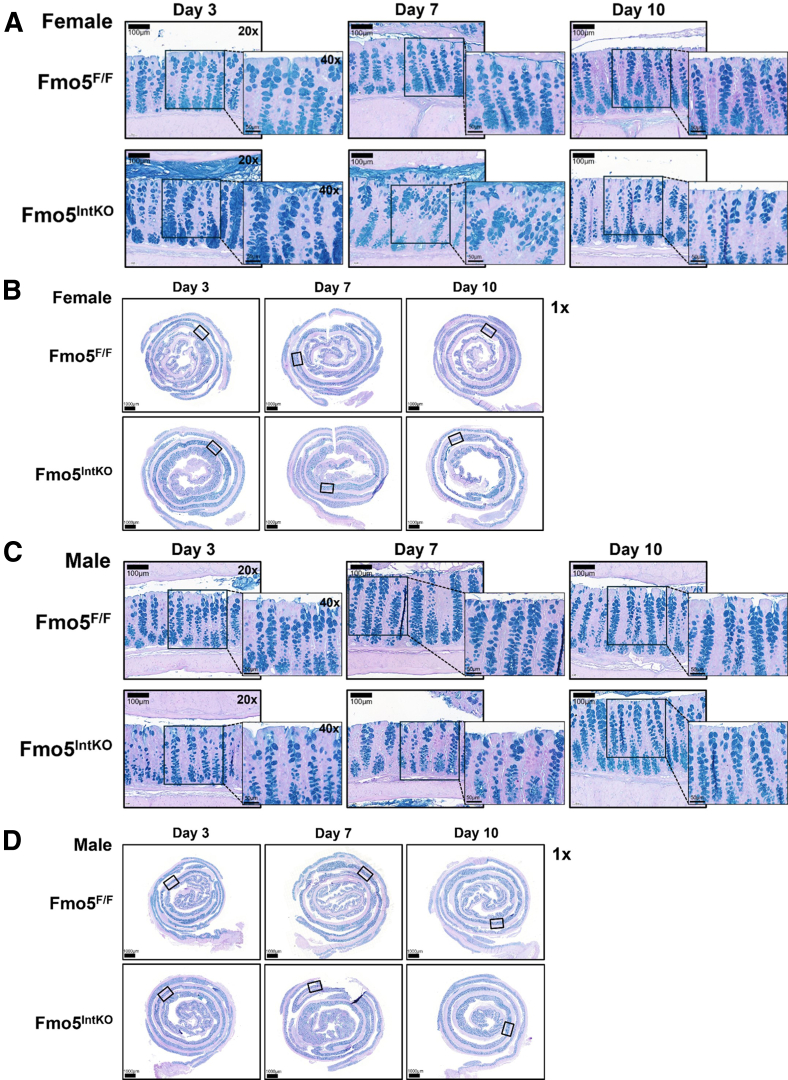
**Time course of GC localization after loss of FMO5 in female mice.** (*A, C*) Representative AB/PAS-stained images of the proximal colon from (*A*) female and (*C*) male Fmo5^F/F^ and Fmo5^IntKO^ mice at days 3-, 7-, and 10- of KO. Scale bar = 100 μm, 20× magnification. Inset images display each boxed area at 40× magnification. Scale bar = 50 μm. (*B, D*) Representative Swiss roll images of whole colons from female (*B*) and male (*D*) Fmo5^F/F^ and Fmo5^IntKO^ mice stained for AB/PAS, corresponding to panels *A* and *C*. Boxes indicate the regions from which the representative images in panels *A* and *C* were taken. 1× magnification, Scale bar = 1000 μm.

To determine if the impaired mucin phenotype developed over time, we assessed mucin staining within crypt GCs in female and male Fmo5^F/F^ and Fmo5^IntKO^ mice at 3, 7, and 10 days of KO (Figure 7*A–D*). We found that female Fmo5^IntKO^ mice had similar proportions of AB/PAS-stained goblet cells within the crypt as their wild-type littermates at day 3 post-tamoxifen treatment (Figure 7*A*). By day 7, female KO mice began to show dysregulated crypt organization, and at day 10 of KO, AB/PAS-stained mucins within GCs in female Fmo5^IntKO^ mice are no longer uniformly spread from the apical to basolateral borders of crypts as in female Fmo5^F/F^ mice (Figure 7*A*). There were no differences in mucus-filled GCs in male Fmo5^IntKO^ mice at day 3 of KO (Figure 7*C*). At day 7 and 10 post-tamoxifen, there remains no obvious difference in AB/PAS-stained GCs within the crypts of male Fmo5^IntKO^ mice when compared with Fmo5^F/F^ (Figure 7*C*).

### Mucus Impairments in Fmo5^IntKO^ Mice Are Not Likely Caused by Dysbiosis

**Figure 8:**
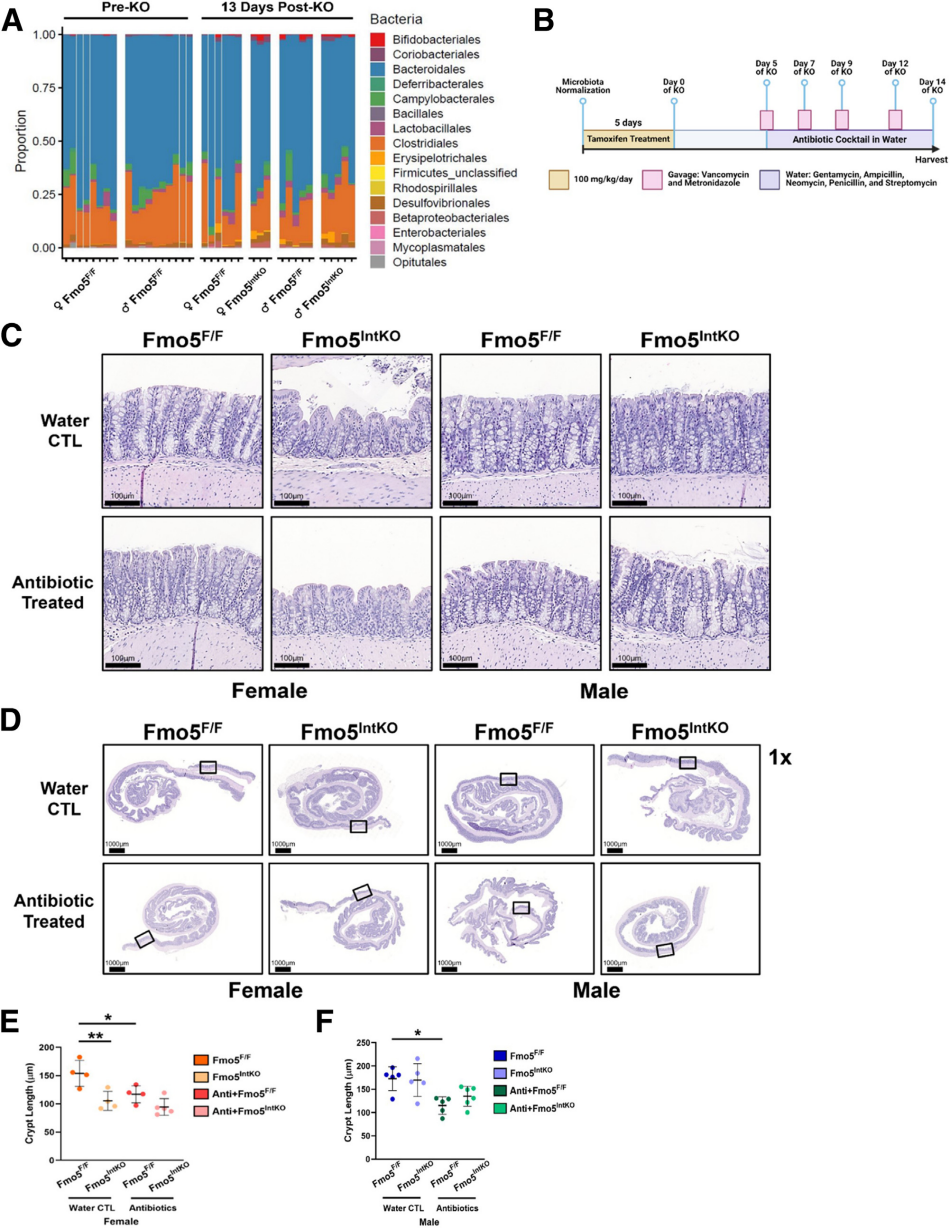
**No evidence of dysbiosis in female Fmo5^IntKO^ mice.** (*A*) Graph displaying bacterial 16s rRNA sequencing of fecal pellets from female and male Fmo5^F/F^ mice prior to tamoxifen treatment, and female and male Fmo5^F/F^ and Fmo5^IntKO^ mice after 13 days of KO (n = 3–6 mice/group). ♀ = female, ♂ = male. (*B*) Experimental design and timeline of tamoxifen and antibiotic treatment. (*C*) Representative images of H&E-stained proximal colons from female and male Fmo5^F/F^ and Fmo5^IntKO^ mice after 14 days of KO. Antibiotic-treated mice were given a mixture of gentamycin, ampicillin, neomycin, penicillin, and streptomycin in drinking water beginning at 5 days post-tamoxifen treatment. Mice were also gavaged with vancomycin and metronidazole (antibiotic-treated) or PBS (water CTL) every other day from 5 to 14 days of KO. 20× magnification, Scale bar = 100 μm. (*D*) Representative Swiss roll images of the proximal colon stained for H&E, corresponding to panel *C*. Boxes indicate the regions from which the representative images in panel *C* were taken. 1× magnification, Scale bar = 1000 μm. (*E, F*) Quantification of crypt length (μm) from (*E*) female and (*F*) male mice described in *C*, where each data point represents the average crypt length of ∼45 to 65 crypts from each individual mouse (n = 4–6 mice/group), and error bars reflect mean ± SD for that mouse. Statistical significance was determined using a nested 1-way ANOVA with Tukey-corrected multiple comparisons. ^∗^*P* < .05, ^∗∗^*P* < .01.

To determine if the damaged epithelium and dysregulated mucus barrier in female Fmo5^IntKO^ mice is due to dysbiosis, we utilized 16s rRNA sequencing of feces from female and male Fmo5^F/F^ and Fmo5^IntKO^ mice. We found no significant changes in the microbial populations of Fmo5^IntKO^ animals compared with their littermate controls after 13 days of KO (Figure 8*A*), indicating that dysbiosis is not likely the primary cause of the intestinal phenotype. Moreover, Fmo5^F/F^ and Fmo5^IntKO^ mice treated with broad-spectrum antibiotics (Figure 8*B*) did not rescue crypt damage or GC alterations in female Fmo5^IntKO^ mice (Figure 8*C–E*). When comparing crypt structure in Fmo5^F/F^ and Fmo5^IntKO^ mice, both wild-type groups and male Fmo5^IntKO^ mice experience crypt shortening with antibiotics (Figure 8*C–F*). In contrast, female Fmo5^IntKO^ crypts do not shorten in response to antibiotics (Figure 8*E*).

### Mucus-specific UPR^ER^ Stress Response Pathway Is Activated in Female Fmo5^IntKO^ Mice

**Figure 9:**
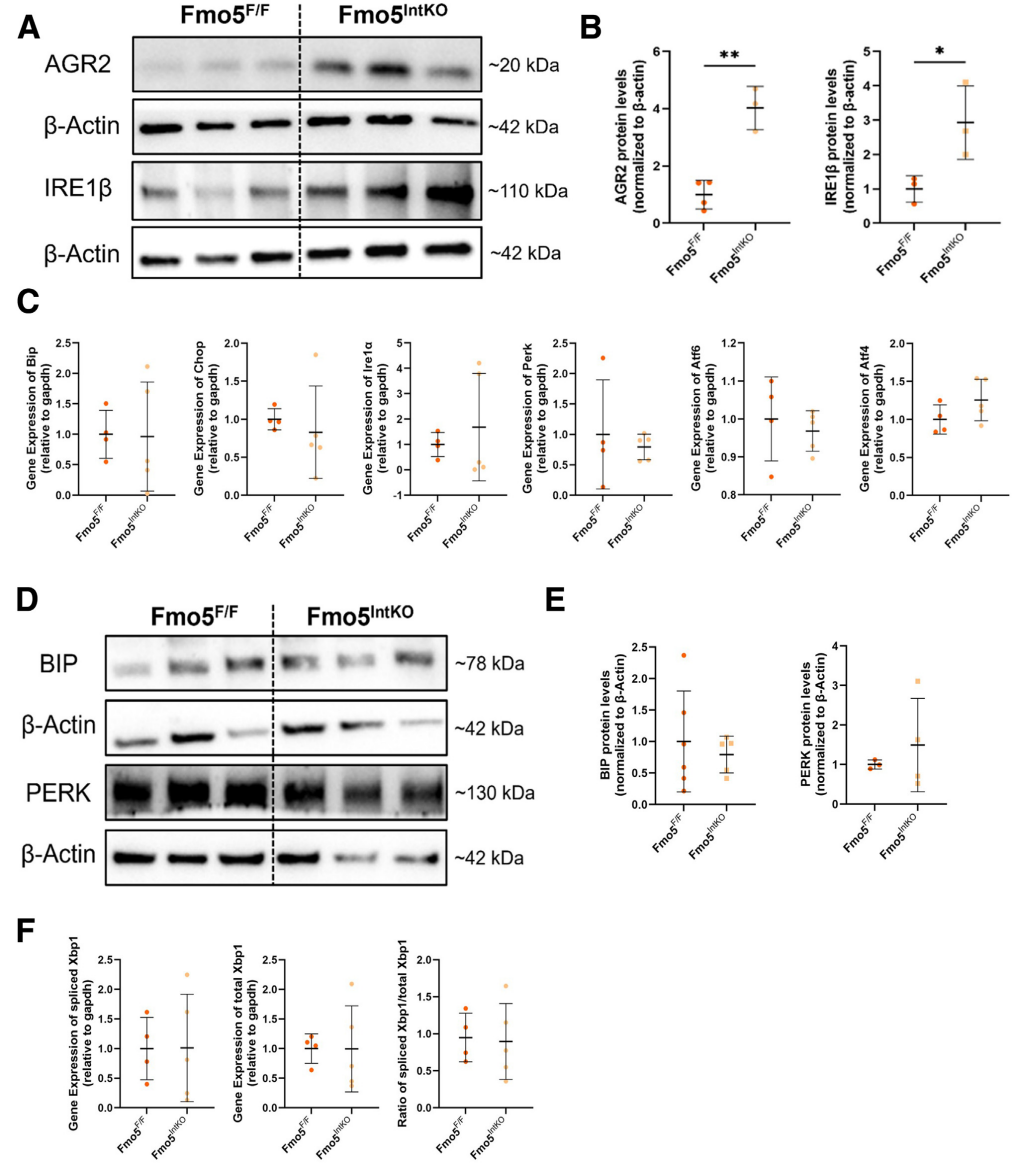
**Mucus-specific UPR^ER^ stress response is activated in female mice without FMO5.** (*A*) Representative Western blots of AGR2 and IRE1β proteins with β-Actin controls from the proximal colon epithelium of female Fmo5^F/F^ and Fmo5^IntKO^ mice at 14 days of KO. (*B*) Quantification of AGR2 and IRE1β protein levels described in *A*, normalized to each sample’s respective β-Actin levels (n = 3–4 mice/group). Lines superimposed on plot display mean ± SD. (*C*) Relative gene expression of canonical ER stress markers *Bip*, *Chop*, *Ire1α*, *Perk*, *Atf6*, and *Atf4* from the colonic epithelium of female Fmo5^F/F^ and Fmo5^IntKO^ mice 14 days after tamoxifen treatment (n = 4–5 mice/group). (*D*) Representative Western blots of BIP and PERK proteins with β-Actin controls from female Fmo5^F/F^ and Fmo5^IntKO^ mice at 14 days of KO. (*E*) Quantification of BIP and PERK protein levels described in *D*, normalized to their respective β-Actin levels (n = 3–6 mice/group). (*F*) Relative gene expression of spliced- and total- *Xbp1* in colon scrapes from female Fmo5^F/F^ and Fmo5^IntKO^ mice 14 days post-tamoxifen treatment (n = 4–5 mice/group). The ratio of spliced *Xbp1* to total *Xbp1* was calculated for each mouse. Plots display mean ± SD for each group. Statistical significance was calculated by unpaired *t*-test in *B*, *C*, *E*, and *F*. ^∗^*P* < .05, ^∗∗^*P* < .01.

The capacity of the ER to properly fold the large MUC2 protein during mucin synthesis is imperative for maintaining a healthy intestinal mucus barrier. Given the critical role of GCs in the complex steps required for mucus production and secretion, their ER has evolved to include an additional, mucus-specific arm of the endoplasmic reticulum unfolded protein response [UPR^ER^], composed of the ER stress sensor inositol-requiring enzyme 1 beta (IRE1β) and its chaperone anterior gradient protein 2 homolog (AGR2).^29^^,^^30^ This specialized UPR^ER^ arm (AGR2 and IRE1β) operates alongside the 3 canonical UPR^ER^ branches IRE1α, protein kinase R-like endoplasmic reticulum kinase (PERK), and ATF6. UPR^ER^ is specifically activated in response to ER stress associated with mucin processing and the high demand for mucus production. We found that AGR2 levels were significantly elevated in Fmo5^IntKO^ mice compared with Fmo5^F/F^ controls (Figure 9*A, B*). This was accompanied by a marked increase in IRE1β protein levels in female Fmo5^IntKO^ mice (Figure 9*A, B*), indicating activation of the mucus-specific ER stress response pathway.

Interestingly, there were no significant differences in the gene expression of key players in the canonical UPR^ER^ stress response (*Bip*, C/EBP homologous protein [*Chop*], endoplasmic reticulum to nucleus signaling 1α [*Ire1α*], *Perk*, activating transcription factor 6 [*Atf6*], activating transcription factor 4 [*Atf4*]) (Figure 9*C*) in female mice disrupted for FMO5, or in binding immunoglobulin protein (BIP) or PERK protein levels (Figure 9*D, E*). In conjunction with this, we see no differences in spliced- or total X-box binding protein 1 (*Xbp1*) expression between female Fmo5^F/F^ and Fmo5^IntKO^ mice (Figure 9*F*), suggesting a lack of increased canonical UPR^ER^ activation in female Fmo5^IntKO^ mice.

### The ER Chaperone Tauroursodeoxycholic Acid Rescues Crypt Dysregulation and Mucus Barrier Impairment in Female Fmo5^IntKO^ Mice

**Figure 10:**
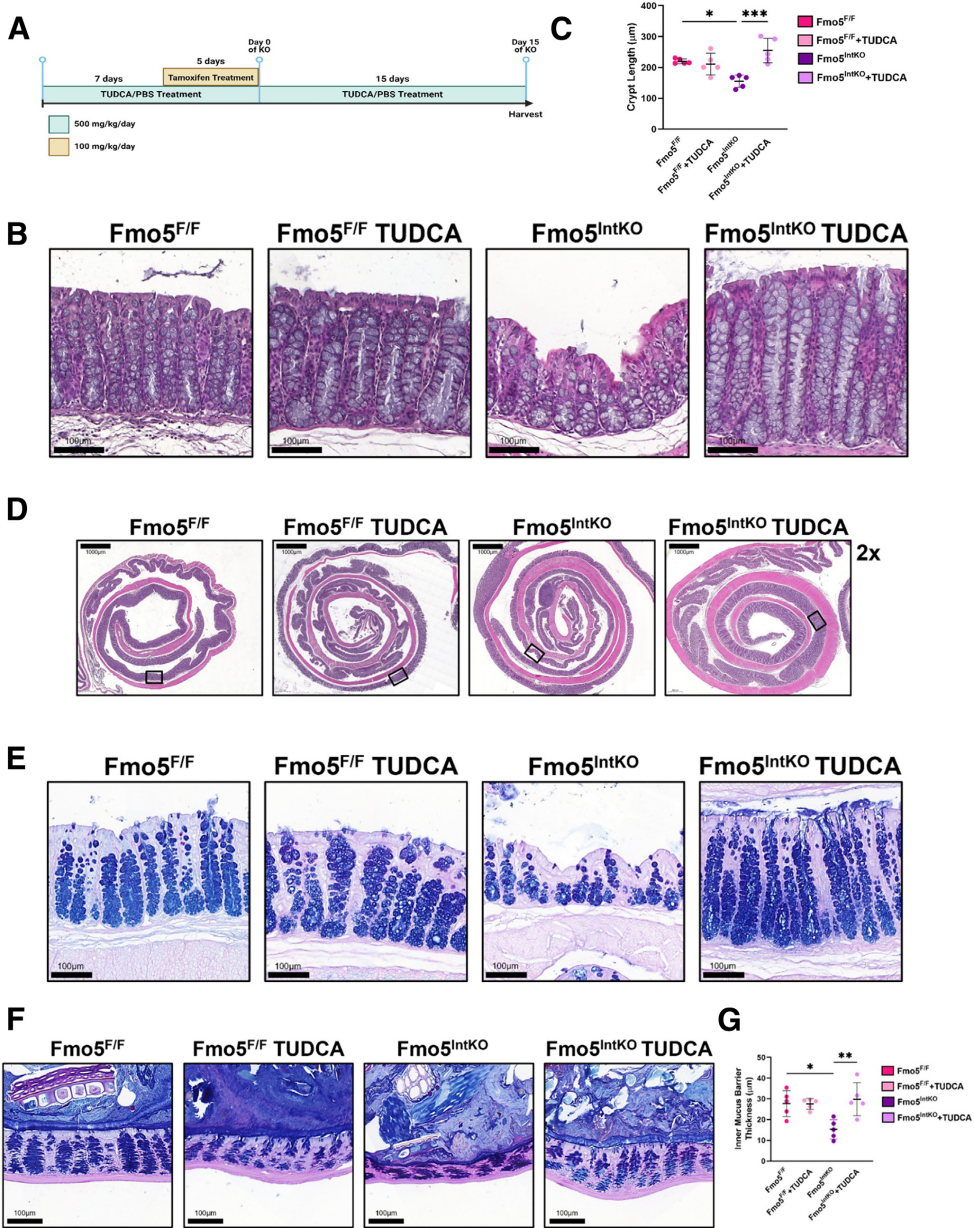
**TUDCA treatment restores crypt length and mucus barrier thickness in female Fmo5^IntKO^ mice.** (*A*) Experimental design and timeline of TUDCA experiment. (*B*) Representative H&E-stained proximal colon from female Fmo5^F/F^ and Fmo5^IntKO^ mice treated with TUDCA or PBS (CTL). 20× magnification, Scale bar = 100 μm. (*C*) Crypt length (μm) quantification of samples represented in *B*. Each data point is an average of 40 to 50 crypt length measurements from each mouse (n = 5 mice/group). Error bars display mean ± SD for each mouse. (*D*) Representative Swiss roll images of the proximal colon stained for H&E, corresponding to panel *B*. Boxes indicate the regions from which the representative images in panel *B* were taken. 2× magnification, Scale bar = 1000 μm. (*E*) AB/PAS-stained images of the proximal colon from female mice ± TUDCA treatment. 20× magnification, Scale bar = 100 μm. (*F*) Representative images of AB/PAS-stained colon cross-sections from TUDCA experiment that were processed using Carnoy’s fixative. 20× magnification, Scale bar = 100 μm. (*G*) Quantification of inner mucus barrier thickness (μm) represented in *F*. Each data point is the average of ∼30 measurements of inner mucus barrier thickness within each mouse, with error bars showing mean ± SD for that mouse (n = 5 mice/group). Statistical significance was calculated by nested 1-way ANOVA with Tukey correction for multiple comparisons (*C, G*). Significant differences between groups are represented by: ^∗^*P* < .05, ^∗∗^*P* < .01, ^∗∗∗^*P* < .001.

To explore if mucus-specific ER stress is contributing to the crypt and mucus barrier defects that we see in female Fmo5^IntKO^ mice, we treated Fmo5^F/F^ and Fmo5^IntKO^ mice with the bile acid chemical ER chaperone tauroursodeoxycholic acid (TUDCA). TUDCA or phosphate-buffered saline (PBS) (control) was given 7 days prior to and during tamoxifen treatment, in addition to 15 days following KO (Figure 10*A*). H&E staining to assess crypt architecture revealed a complete rescue of crypt length in female Fmo5^IntKO^ mice with TUDCA treatment, compared with Fmo5^IntKO^ mice given PBS (Figure 10*B–D*). AB/PAS staining demonstrated TUDCA treatment restored mucin levels in GCs within the crypt. Female Fmo5^IntKO^ mice treated with TUDCA had visually comparable, if not potentially increased, mucin levels compared with controls (Figure 10*E*). Additionally, we found that TUDCA treatment in Fmo5^IntKO^ mice completely restored inner mucus barrier thickness when compared with Fmo5^IntKO^ mice without TUDCA (Figure 10*F, G*). The ability of an ER chaperone to improve crypt and mucus defects in mice without FMO5 suggests that the underlying cause of these defects involves protein folding and/or the ER stress response.

## Discussion

Together, our results support a model in which FMOs play a conserved, integral role in maintaining intestinal homeostasis. In *C. elegans,* we show that *fmo-2* is necessary to maintain structural integrity and barrier function in the intestine (Figure 1). Building upon this, to define the conserved role of FMOs in maintaining intestinal homeostasis, we generated a tamoxifen-inducible, intestine-specific mouse model of mammalian FMO5. Characterization of this mouse line revealed a sex-dependent role for FMO5 in the maintenance of epithelial architecture in the colon (Figure 2*D*). Our results also demonstrate the necessity of FMO5 for GC localization and MUC2 expression (Figures 4*C* and 5*B*), leading to thinning of the mucosal barrier with loss of FMO5 in mice (Figure 6*C, D*). High demand on the GC ER to process mucins and evidence of mucus-specific ER stress in Fmo5^IntKO^ animals led us to hypothesize that FMO5 is essential to maintain ER homeostasis during mucus production. This hypothesis is supported by the rescue of crypt architecture and mucus barrier thickness with the addition of the bile acid ER chaperone, TUDCA (Figure 10*B, F*). Our results (1) support a new role for FMO5 in maintaining intestinal ER homeostasis, and (2) introduce a new mouse model of intestinal dysfunction. Overall, this work highlights the importance of FMO5 in maintaining a healthy intestine in mice.

Our results show the highly conserved enzyme, FMO5, when disrupted in mice, exhibits many characteristics of chronic gastrointestinal tract (GI) disease, but only in female mice. This is a striking sex difference that parallels that observed in humans, despite no clear linkage between FMO5 and GI disease pathogenesis. The mechanism underlying sexual dimorphism in GI disease is largely unclear, and this model provides a unique opportunity within the field to dissect the cellular mechanisms. Both the prevalence and progression of IBD differs in humans based on sex, where women are more likely to develop Crohn’s Disease (CD) and men are more susceptible to UC.^22^ One study found that post age 45, females are up to 32% less likely to be diagnosed with UC when compared with males.^31^ This is coupled with another report showing that *FMO5* expression was down-regulated in a UC dataset.^32^ Interestingly, we found no differences in *Fmo5* expression levels in the intestinal epithelium between female and male mice at 1, 3, or 6 months of age (Figure 2*G*). This finding does not support the hypothesis that female mice are more susceptible to FMO5 loss due to higher intestinal FMO5 levels. We speculate that FMO5 may interact with sex hormones to provide protection from intestinal dysfunction in female mice.

Maintaining the physical integrity of the intestine by epithelial cells is critical for preserving intestinal homeostasis and function. Morphologic assessment of crypt architecture is a useful tool when diagnosing GI disease in a clinical setting, and its relevance to disease spans into mammalian models.^33^^,^^34^ Studies show that shortening of crypts in the colon (crypt atrophy), crypt distortion, and crypt loss are all present in mouse models of IBD, and these alterations have a direct impact on epithelial health.^35^ Additionally, crypt shortening and disarray along with crypt branching and mucin depletion are diagnostic features of UC in humans.^36^, ^37^, ^38^, ^39^ These characteristics are observed in the colonic epithelium of female Fmo5^IntKO^ mice, leading us to hypothesize that FMO5 plays a protective role in GI disease onset. The severity of crypt shortening that we report (Figure 2*D, E*) is comparable to mice following acute colitis.^35^ Crypt lengthening in male Fmo5^IntKO^ mice could be attributed to crypt epithelial hyperplasia or crypts elongating to increase cell number and surface area to maintain epithelial function in Fmo5^IntKO^ mice (Figure 3*D, E*).^40^^,^^41^ We also found that the loss of structural homeostasis in the colon of female Fmo5^IntKO^ mice compounds with each epithelial turnover, resulting in dramatic crypt distortion by 14 days post-KO (Figures 2*D*; 3*A*). We speculate that the dysregulation of crypt architecture in female Fmo5^IntKO^ mice at day 10 of KO (Figures 3*A*; 7*A*) is a result of compensatory effort to maintain epithelial homeostasis. By day 14 of KO (Figures 2*D*; 6*A*), these mice lose the ability to combat the progressive impairments resulting from FMO5 loss. Interestingly, there is a lack of distinct differences in crypt structure and AB/PAS-stained GCs between Fmo5^F/F^ and Fmo5^IntKO^ mice at day 3 of KO (Figures 3*A, D*; 7*A, C*). Considering the colonic epithelium is completely replaced every 3 to 5 days in mice,^42^ these findings indicate that, although pre-existing epithelial cells are largely unaffected by FMO5 loss, subsequent epithelial turnover is impaired in the absence of FMO5. From this, we conclude that FMO5 is necessary for the formation of a healthy epithelium in the colon. The absence of further crypt shortening in female Fmo5^IntKO^ mice with antibiotic treatment could be due to the loss of beneficial bacteria in the gut. We speculate that female Fmo5^IntKO^ mice are severely compromised, and that any greater loss of crypt architecture will be detrimental to survival. Despite the lack of evidence linking the Fmo5^IntKO^ phenotype to dysbiosis, it is possible that female Fmo5^IntKO^ mice have different interactions with homeostatic gut bacteria. This possibility is supported by published work on a FMO5 constitutive KO animal model, where male mice were suggested to respond differently to microbiota.^43^ Additional investigation is needed to determine if female mice without FMO5 are hypersusceptible to intestinal cues or intestine-specific stress.

Studies show that altered epithelial architecture in mice can result from defects in cell differentiation and migration, where cells are unable to terminally differentiate or effectively migrate up the crypt toward the lumen of the intestine.^25^ We find that GCs in female Fmo5^IntKO^ mice are clustered toward the bottom of the crypt rather than being spread uniformly throughout (Figure 4*C*). This is intriguing, as mice lacking the primary colonic GC mucin (MUC2) exhibit altered crypt morphology and have impairments in GC migration up the crypt. These data are consistent with those from Scott et al, who observed decreased GC number in constitutive, whole body male Fmo5^-/-^ mice.^43^ It remains unclear if the dysregulated crypt architecture and decreased crypt length in female Fmo5^IntKO^ mice result from a loss of GCs in the upper crypt or if the lack of crypt structure disrupts proper GC migration and crypt formation. Previous studies in mouse models with missense mutations in MUC2 (Winnie^44^ and Eeyore^44^), or in MUC2^-/-^ mice^45^ report decreased GC numbers in the proximal colon, despite crypt lengths being maintained or even increased,^44^^,^^45^ suggesting that GC loss can occur independently of changes in crypt length. In contrast, another study reported a correlation between reduced GC numbers and more pronounced disruption of crypt architecture in MUC2^-/-^ mice.^5^ Further investigation is needed to determine which of these mechanisms contribute to the FMO5 knockout phenotype in female mice.

Multiple factors necessary to drive GC maturation and migration have been identified, and several studies link impairments in these with the development of IBD.^39^^,^^46^ One phenotype specifically attributed to cases of UC in humans, rather than IBD as a whole, is a loss of mucin-producing GCs in the top half of the crypt coupled with depleted mucus barrier.^37^^,^^47^, ^48^, ^49^ Additionally, GC clustering and loss of upper crypt GCs in unchallenged female Fmo5^IntKO^ mice (Figure 4*C*) is consistent with published models of DSS-induced colitis, where GC dysfunction is attributed to decreased GC maturation.^39^^,^^46^ The lack of differences in absorptive and secretory progenitor cell expression in our mice (Figure 5*E*) rule out the possibility that impaired mucus production in female Fmo5^IntKO^ mice is caused by decreased secretory cell differentiation. This further supports the hypothesis that maturation is the likely GC defect occurring in female Fmo5^IntKO^ mice. These findings, coupled with an increase in GC gene expression (*Muc2*, *Tff3*) that we suspect is a compensatory increase in response to impaired mucus barrier formation (Figure 4*A*), suggest that FMO5 contributes to GC regulation in female mice.

Maintaining a protective mucus barrier is the culmination of a range of structural, functional, and biochemical processes. Here, we show that intestinal FMO5 is necessary for proper mucus production within colonic crypts and mucus barrier maintenance in female mice (Figure 5*A, C*). Previously published studies show that impairments in MUC2 folding, defects in O- and N-glycosylation, and aggregation of the non-glycosylated form of MUC2 in mice each cause hyper-susceptibility to colitis coupled with aberrant GC function.^50^ It is likely that one or more of these protein processing impairments occur in Fmo5^IntKO^ females, given that: (1) FMO5 resides on the ER membrane; and (2) we find evidence of mucus-related ER stress. A previous study showed that the BIP/IRE1α arm of the UPR senses a wide variety of ligands that cause ER stress but does not respond to excess or misfolded MUC2 presence.^29^ The mucin-sensitive UPR^ER^ arm made up of AGR2 and IRE1β is hypothesized to take on the responsibility for the proper folding of mucins in GCs.^29^^,^^51^ This may prevent the canonical ER stress response from overwhelming the ER in mucosal epithelial cells. Our findings are consistent with this, as we show increased AGR2 and IRE1β protein levels in female Fmo5^IntKO^ mice (Figure 9*A, B*), in the absence of typical UPR^ER^ activation (Figure 9*C–F*). Complete rescue of crypt architecture and mucosal barrier impairments in female Fmo5^IntKO^ mice with the addition of the ER chaperone TUDCA^52^ (Figure 10) implicates protein processing dysfunction in the ER of these mice. This finding leads us to speculate if FMO5 loss causes an accumulation of MUC2 proteins in the GC ER that are unable to fold correctly, triggering AGR2 and IRE1β activation. It is also possible that GCs in female Fmo5^IntKO^ mice have increased sensitivity to ER stress, which could increase their susceptibility to ER stress-induced damage. Although further exploration of this system is necessary to identify the exact molecular mechanism of how FMO5 plays into ER stress regulation, our results imply that GC and mucus barrier defects in female Fmo5^IntKO^ mice are caused by altered protein folding and/or ER stress. This establishes a role for FMO5 in maintaining mucosal barrier integrity and intestinal homeostasis in mice through ER stress protection.

Together, this work describes a previously unidentified role for FMO5 in maintaining intestinal integrity and mucosal barrier homeostasis. There remains a need to identify the cellular mechanism(s) by which acute loss of FMO5 leads to defects in GC maturation and migration, and if an altered metabolic environment is involved. Additional investigation is also warranted to determine if there are any changes to the biochemical components of mucus produced in mice without FMO5, and how those changes influence mucus barrier integrity and resilience. Future directions for this project include clarifying the cellular mechanism of how intestinal FMO5 influences GI disease onset, with the overall goal to utilize the resulting data to improve therapeutic treatment for GI diseases.

## Materials and Methods

### Worm Strain and Maintenance

Wild-type N2, KAE9 (*fmo-2* OE), VC1668 (*fmo-2* KO), ELT-60 (ACT-5::green fluorescent protein [GFP]), and LZR1 (*fmo-2p::mCherry* [*MC*]) strains of *C. elegans* were age synchronized by timed egg lay prior to use. Strains were grown and maintained on solid nematode growth media (NGM) throughout their lifespan. The nematodes were fed *E. coli* OP50 throughout life except where RNAi (*E. coli* HT115 *fmo-2* and Empty Vector Control) were fed instead. All experiments were performed at 20°C.

### Smurf Assay in *C. elegans*

Wild-type, *fmo-2* KO, and *fmo-2* OE worms were synchronized and fed *E. coli* OP50 bacteria dyed with 0.10% wt/vol. Erioglaucine disodium salt (Sigma Aldrich, Federal food, drug, and cosmetic dye [FD&C]). On each day of analysis, worms were placed on a 3% agarose pad on a glass slide, and 1 M sodium azide was used to induce paralysis before imaging using a Leica fluorescent microscope. Fluorescent intensity of each worm was quantified using ImageJ.

### DSS Assay in *C. elegans*

*fmo-2p::mCherry* worms were age synchronized and fed with *E. coli* OP50 until day 1 of adulthood. They were then incubated in M9 wash solution with OP50 ± 5% DSS for 20 hours at 20°C prior to imaging. Worms were placed on a 3% agarose pad on a glass slide, and 1 M sodium azide was used to induce paralysis before imaging using a Leica fluorescent microscope. Fluorescent intensity of each worm was quantified using ImageJ.

### Mouse Line Generation

The Fmo5^F/F^ mouse line was generated in collaboration with the Transgenic Animal Model Core of the University of Michigan’s Biomedical Research Core Facilities. Briefly, purified DNA was microinjected into fertilized eggs obtained by mating (C57BL/6 X SJL) F1 or C57BL/6 female mice with (C57BL/6 X SJL) F1 male mice. Pronuclear microinjection was performed as described.^53^ Resulting Fmo5^loxP/loxP^ mice were genotypically verified before being crossed into the intestine-specific, inducible VillinER^T2^ cre mouse line and backcrossed a minimum of 5 generations prior to using for experiments.

### Mouse Husbandry

All mice used in this study were 8- to 12-week-old VillinER^T2^ cre^+^; Fmo5^loxP/loxP^ (Fmo5^IntKO^) and Fmo5^loxP/loxP^ (Fmo5^F/F^) female and male mice in a C57BL/6 background. Littermate controls were used whenever possible. All mice were housed in the pathogen-free Unit for Laboratory Animal Management at the University of Michigan. Mice were kept under a 12-hour light/12-hour dark cycle, and standard chow and water were provided *ad libitum.* All animal studies were conducted in accordance with Association for Assessment and Accreditation of Laboratory Animal Care International guidelines and approved by the University Committee on the Use and Care of Animals at the University of Michigan.

### Tamoxifen Treatment

Mice were given intraperitoneal injection of 100 mg/kg tamoxifen^54^, ^55^, ^56^ (MP Biomedical) in corn oil once daily for 5 days. Mice were weighed daily during treatment to monitor health and were housed by genotype and sex. The 5th and final day of injection was considered day 0 of FMO5 knockout for all experiments in this study. Both female and male mice were used for each experiment except for Figure 5*B* and *F*, Figure 9, and Figure 10, where only female mice were used. Over the course of the study, we observed a phenotype that was sex-dependent, and the listed experiments were primarily relevant to the phenotype observed in female mice.

### Histology

To prepare samples for general histological analysis, the colon was removed at necropsy and opened lengthwise. The colon was rinsed in 1 × PBS and rolled to create a Swiss roll.^57^ Samples were incubated in 10% buffered formalin phosphate (Fisher Chemical) overnight at room temperature (RT) before being moved to 70% ethanol until further processing. Tissues were dehydrated by paraffin wax embedding and sliced into 4-μm sections.^58^ All images taken from Swiss rolls throughout this work for quantification and representation were taken in the section of the colon that is distal to the folds found in the proximal colon, but proximal to the overall midpoint of the colon. Swiss rolls shown in Figure 3*C* and *F* and Figure 7*B* and 7*D* display the entire colon rolled. Swiss rolls shown in Figure 2*F*, 5*C*, 6*B*, and 10*D* display only the proximal half of the colon rolled. Images were taken in areas where the lamina propria was directly in contact with the epithelium, and areas that displayed a transverse view of crypts were avoided for imaging.

For mucus barrier histologic analysis, upon euthanasia, the colon was removed and kept intact. Horizontal cuts were made above and below a fecal pellet midway down the colon, and the sample was immediately placed into Carnoy’s fixative (Spectrum) to preserve the mucus barrier.^59^ Samples were incubated overnight at RT in Carnoy’s solution prior to being moved to 70% ethanol and stored at 4°C until paraffin embedding. Embedding and sectioning was performed using the same method as described above.

### Immunofluorescence

Paraffin sections were deparaffinized using xylene and rehydrated using washes of 100%, 95%, and 80% ethanol at RT. Samples underwent antigen retrieval in sodium citrate buffer with a pH of 6.0 (Thermo Scientific) at 100°C for 20 minutes. Samples were blocked with goat serum and incubated in MUC2 primary antibody (1:1000, Proteintech) overnight at 4° at a concentration of 1:1000. The following day, slides were rinsed and incubated in secondary antibody (Anti-GFP, Invitrogen) for 1 hour at RT at 1:500 concentration. Following incubation, samples were washed, and cover slips were mounted using Prolong Gold with 4′,6-diamidino-2-phenylindole (DAPI) (Fisher Chemical). Fluorescent images in Figure 4*C* and Figure 5*F* were taken on a Nikon Ti2 Widefield microscope. Images in Figure 5*B* were taken on a Confocal LSM 800 microscope.

### EdU Injection and Staining

Mice were given intraperitoneal injection of 25 mg/kg of EdU (Invitrogen) dissolved in 1 × PBS on day 13 (24-hour experiment) and day 14 (2-hour experiment) of KO. Exactly 2 and 24 hours later, mice were euthanized by C0_2_ inhalation, and intestinal tissue was collected for histologic analysis. Paraffin sections were deparaffinized using xylene and rehydrated using washes of 100%, 95%, and 75% ethanol at RT. IF staining (24-hour experiment only) as described above was performed until after the secondary incubation step. Slides were then incubated in 0.5% Triton X-100/PBS for 20 minutes at RT, and EdU staining was performed using a Click-iT EdU Kit (Invitrogen) following the included protocol. Briefly, slides were incubated in fresh Click-iT reaction cocktail for 30 minutes at RT in the dark. Coverslips were mounted using Prolong Gold with DAPI (Fisher Chemical).

### Antibiotic Treatment

Prior to tamoxifen treatment, 8- to 12-week-old female and male Fmo5^F/F^ and Fmo5^IntKO^ mice underwent microbiota normalization by collecting, mixing, and redistributing the bedding of all cages involved in the experiment every other day for 2 weeks. Following microbiota normalization, mice were given intraperitoneal injections of tamoxifen (100 mg/kg of body weight, MP Biochemical) for 5 days. From days 5 to 14 of KO, mice were provided an antibiotic cocktail or water (control) in their water supply. The antibiotic treatment consisted of gentamycin (500 mg/L), ampicillin (1 g/L), neomycin (1 g/L), penicillin (100 U/L), and streptomycin (200 mg/L). Mice were also given a 150-μL gavage consisting of vancomycin (1 mg/mL) and metronidazole (0.5 mg/mL) or 1 × PBS (control) every other day starting on day 5 of KO. Mice were euthanized at day 14 of KO, and proximal colon samples were collected for histologic analysis.

### 16s rRNA Sequencing

Sequencing of bacterial 16s rRNA was performed using the Microbial Systems Molecular Biology Lab, which is part of the University of Michigan Host Microbiome Initiative. Eight- to 12-week-old mice were given intraperitoneal injection of 100 mg/kg tamoxifen (MP Biomedical) for 5 days. Baseline fecal pellets were collected from mice prior to tamoxifen treatment. After 13 days of KO, mice were necropsied, and fecal pellets were collected from the intestines. Using a dual index sequencing strategy, the V4 regions of the 16s rRNA was amplified. The samples were then sequenced using MiSeq Reagent kit V2 500 cycles in Illumina MiSeq. Mothur (v1.42.3) was used to analyze bacterial communities, and quality of the samples was demonstrated via aligned reading of a DNA extraction control and genotype clustering via principal component analysis.

### Real-time Quantitative Polymerase Chain Reaction

RNA was extracted for gene expression analysis from colon epithelial scrapes using the chloroform method. Samples were homogenized in Trizol (Ambion), the supernatant was removed, and chloroform (Sigma-Aldrich) was used to cause phase separation. Isopropanol (Acros Organics) was added to cause RNA precipitation before the pellet was washed in 70% ethanol and allowed to dry before being reconstituted with water. RNA concentrations were measured by Nanodrop and synthesized into cDNA by Reverse Transcription. One μg of RNA was loaded into each reaction including Oligo(dT) (Promega), dNTP mix (Thermo Scientific), and sterile water. Samples were heated at 65°C for 6 minutes and quick chilled on ice prior to adding First Strand Buffer (Invitrogen) and DTT (Invitrogen) and incubating at 42°C for 2 minutes. SuperScript II RT (Invitrogen) was added to each reaction and incubated at 42°C for 50 minutes prior to enzyme inactivation by heating at 70°C for 15 minutes. Real-time quantitative polymerase chain reaction (RT-qPCR) was performed using SYBR green master mix (Applied Biosystems), sample cDNA, and forward and reverse primers. qPCR cycles were run at a standard ramp speed with each sample run in technical duplicate or triplicate. The resulting comparative ΔΔCT values were used to calculate gene expression between samples with technical replicates averaged.

### H&E Staining

Paraffin sections were deparaffinized using xylene (Acros Organics) and rehydrated using washes of 100%, 95%, and 70% ethanol at room temperature. Slides were stained in Hematoxylin (Gill’s 2×) (RICCA) for 2 minutes and dipped 1 to 2 times in Bluing Reagent (ScyTek Laboratories). The slides were next counterstained in eosin and dehydrated using washes of 95% ethanol, 100% ethanol, and xylene. Coverslips were mounted onto slides using xylene-based Permount (Fisher Chemical).

### AB/PAS Staining

Paraffin sections were deparaffinized using xylene (Acros Organics) and rehydrated using washes of 100% and 95% ethanol at RT. Samples were incubated in 3% acetic acid for 3 minutes followed by Alcian Blue (Newcomer Supply) for 30 minutes at room temperature. Incubation in 0.5% periodic acid (Alfa Aesar) for 10 minutes was used to oxidize samples. Slides were next incubated in Schiff’s Reagent (Electron Microscopy Sciences) at room temperature for 30 minutes. Samples were dehydrated using washes of 95% ethanol, 100% ethanol, and xylene. Coverslips were mounted onto slides using xylene-based Permount (Fisher Chemical).

### Western Blot

Upon necropsy, proximal colon epithelium was harvested and flash frozen in liquid nitrogen. Protein was extracted using RIPA buffer (Thermo Fisher) with protease and phosphatase inhibitors (Thermo Fisher). Protein concentration was quantified using a Pierce BSA protein assay kit (Thermo Fisher) and normalized, and samples for Western blot were prepared using RIPA buffer and loading dye to a final concentration of 4×. Samples were incubated at 95°C for 5 minutes prior to being flash frozen in liquid nitrogen and stored at −20°C until use. Six μg of protein from each sample was loaded into a 4% to 20% Mini-PROTEAN® TGX gel (BioRad), and proteins were separated by size using electrophoresis. Gels were transferred onto a 0.45-μM nitrocellulose membrane by wet transfer method. Membranes were blocked in 5% milk for 1 hour at RT prior to incubation with primary antibodies (IRE1β: 1:500 and AGR2: 1:1000 from Invitrogen, and FMO5: 1:1000 from Cell Signaling) overnight at 4°C. The following day, membranes were washed and incubated in anti-rabbit secondary antibody (Cell Signaling) at a concentration of 1:2000 in 5% milk for 1 hour at RT. Proteins were visualized using an ECL kit (Thermo Fisher) and Chemidoc Imager before protein levels were analyzed using ImageJ.

### TUDCA Treatment

Mice were given intraperitoneal (IP) injection of 500 mg/kg/day of TUDCA (Cayman Chemical) dissolved in 1 × PBS once daily starting 7 days prior to tamoxifen treatment. TUDCA treatment was continued during the tamoxifen treatment regime, where mice received an IP injection of each chemical for 5 days. Following the conclusion of tamoxifen treatment, mice received TUDCA injections for an additional 14 days and were euthanized at 14 days of KO.

### Statistical Analysis

All data are displayed as mean ± standard deviation. The sample size of animals in each experiment are in each figure legend, where “n” represents independent biological replicates. All plots were graphed, and statistical tests were calculated using GraphPad Prism Version 10.2.2. Statistical significance for all comparisons between 2 experimental groups were calculated using 2-tailed, unpaired *t*-tests. Statistical significance for all comparisons with greater than 2 experimental groups was calculated in using 1-way analysis of variance (ANOVA) (or nested 1-way ANOVA) with multiple comparisons and Tukey post-hoc corrections when appropriate. Statistical significance was determined for *P* values less than .05, and are reported as ^∗^ when *P* < .05, ^∗∗^ when *P* < .01, ^∗∗∗^ when *P* < .001, and ^∗∗∗∗^ when *P* < .0001. For graphs displaying multiple measurements for each independent mouse (crypt length and inner mucus barrier thickness), each plot represents the measurements from a single mouse with mean and standard deviation (SD) values for that mouse superimposed on the plot. Experimental design images and the image in Figure 1*A* were made with Biorender. All authors had access to study data and have reviewed, edited, and approved the final manuscript.
